# The Prevalence, Characteristics, and Putative Mechanisms of Dual Antigen-Positive Membranous Nephropathy: The Underestimated Condition

**DOI:** 10.3390/ijms25115931

**Published:** 2024-05-29

**Authors:** Takahiro Uchida, Takashi Oda

**Affiliations:** Department of Nephrology and Blood Purification, Kidney Disease Center, Tokyo Medical University Hachioji Medical Center, Hachioji 193-0998, Japan; tu05090224@gmail.com

**Keywords:** dual antigen-positive, membranous nephropathy, nephrotic syndrome, neural epidermal growth factor-like 1, podocyte phospholipase A2 receptor, thrombospondin type-1 domain-containing 7A

## Abstract

Following the discovery of podocyte phospholipase A2 receptor and thrombospondin type-1 domain-containing 7A, various potential target antigens for membranous nephropathy (MN) have been reported one after another. MN target antigens have now been identified in a significant proportion of patients, and a new classification framework classifies patients with MN based on the detected antigen and associated disease phenotype. A serology-based approach that does not require a histological diagnosis for patients suspected of having MN has also been proposed. However, there have been cases in which dual positivity for MN antigens and/or corresponding antibodies has been shown. Importantly, some of them showed a transition of the affected patient’s immune responses to MN antigens, suggesting that serological diagnosis changes depending on the timing of the analysis. In this review, we provide detailed information on these cases and present an overview of our recent understanding of their putative mechanisms involved in these cases. Greater awareness is required to adequately recognize and develop appropriate therapeutic strategies for this condition.

## 1. Introduction

Membranous nephropathy (MN) is a leading cause of nephrotic syndrome in adults and has been conventionally classified into two groups: the idiopathic type without an underlying disease, and the secondary type, which reportedly accounts for approximately 20% of patients with MN and is associated with causative systemic diseases, such as autoimmune disease, malignancy, infection, or drug allergies [1,2,3]. Immunosuppressive therapy is often required in cases of idiopathic MN, whereas the management of secondary MN is based on the treatment of the underlying primary illness.

The discovery of the podocyte phospholipase A2 receptor (PLA_2_R) in 2009 resulted in drastic changes in the approach to MN [4]. The detection of serum anti-PLA_2_R antibodies has been shown to have both high sensitivity and specificity for the diagnosis of MN [5], and antibody levels are reportedly correlated with disease activity [3]. Thus, renal biopsy is not routinely required to confirm the diagnosis of MN in patients with nephrotic syndrome and positive results for serum anti-PLA_2_R antibodies [3,6]. Following the discovery of PLA_2_R, thrombospondin type-1 domain-containing 7A (THSD7A) was identified as the second antigenic target of MN in 2014 [7]. PLA_2_R-positive MN and THSD7A-positive MN account for 50–80% and 1–5% of cases with MN, respectively [8,9]. Both PLA_2_R and TSHD7A are endogenous antigens that are expressed on podocytes, and circulating autoantibodies against them can be detected in the serum of patients using Western blotting, indirect immunofluorescence (IF), or enzyme-linked immunosorbent assay (ELISA) and are shown to belong to the immunoglobulin (Ig)G4 subclass. Both IF and immunoperoxidase staining of the patients’ renal tissues demonstrated the presence of these antigens within the glomerular subepithelial immune complexes. In evaluating immunoperoxidase staining, however, it is important to distinguish between normal weak staining in podocytes (as these are endogenous antigens normally present in podocytes) and distinct granular positive staining within immune complexes in glomerular subepithelial sites. The normal staining pattern of endogenous podocyte antigens has been previously described [10,11].

In the last 5 years, potential target antigens for MN have been identified one after another [8,12]. Most of these antigens have been identified via laser capture microdissection of glomeruli, followed by proteolytic digestion and tandem mass spectrometry (LCM/MS) [13]. Consequently, target antigens have been reportedly identified in 80–90% of patients with MN [8]. These advances in antigen knowledge have led to the proposal of a new classification of MN that classifies patients based on the detected antigen and the associated disease phenotype [8,12]. This classification is based on the concept that the antigens are mutually exclusive; that is, only one target antigen is detected in each patient. 

However, several cases have been reported in which dual positivity in MN antigens and/or the corresponding antibodies was observed. The precise mechanisms remain unclear and may be just coincidental; however, it is suggested that some immune responses occurring during the disease processes, which target the “first” MN antigen, induce abnormal exposure of the “second” podocyte antigen and damage to the glomerular basement membrane, leading to the development of second autoantibody and its glomerular deposition [14,15]. 

We came to have the research question of “What are the clinical features of cases of dual antigen-positive MN as compared with single antigen-positive or negative MN cases?” To resolve this question, we surveyed articles on cases with dual antigen-positive MN using PubMed as the database and “dual antigen” and “MN” as the key words. The main purpose of this review article is to increase awareness of dual antigen-positive MN, which could be an underestimated condition.

## 2. Characteristics of MN with Dual Antigen Positivity

The characteristics of the target antigens reportedly involved in dual antigen-positive MN cases are summarized in Table 1.

There have been several reports of dual-positive cases for PLA_2_R and THSD7A (8 Refs) [11,16,17,18,19,20,21,22]. Colocalization of PLA_2_R and THSD7A in immune deposits has been observed via confocal microscopy in some cases, suggesting that both antigens form an immune complex [16]. PLA_2_R and THSD7A share a common epitope motif in the N-terminal region that may induce the production of autoantibodies directed against both or predominantly one of these antigens [20,23]. However, the circulating levels of these autoantibodies evaluated using ELISA are reportedly very different between patients; some are positive for both antigens, whereas others are positive only for PLA_2_R [17]. Therefore, the pathogenesis in these dual antigen-positive patients remains unclear.

Neural epidermal growth factor-like 1 protein (NELL1) is now considered the second most common antigen, accounting for approximately 10% of patients with MN [8,24]; it is reportedly not expressed in podocytes, suggesting that it is secreted and deposited in the glomeruli. Serum anti-NELL1 antibodies are detected in patients with NELL1-associated MN [25]. A recent interesting study, which performed immunostaining for PLA_2_R, THSD7A, and NELL1 in the renal tissue and evaluated the corresponding circulating antibodies, reported the clinicopathological information of “dual antigen-positive” MN [26]. In the study, the proportion of patients with MN showing dual antigen positivity, defined as positive glomerular deposits and/or circulating antibodies, was approximately 0.7%, and these cases consisted of “PLA_2_R and THSD7A dual-positive cases” and “PLA_2_R and NELL1 dual-positive cases”. Compared to patients with PLA_2_R single-positive MN, those with dual antigen-positive MN had a higher renal tissue IgG1 positivity rate, and the time required to achieve remission was longer. The authors also conducted a literature review and reported that the proportion of dual antigen-positive MN ranged from 0.2% to 2.8%.

MN is sometimes observed along with myeloperoxidase (MPO)-anti-neutrophil cytoplasmic antibody (ANCA)-associated glomerulonephritis; however, some consider MN and ANCA-associated glomerulonephritis to be two mutually independent diseases [27]. In contrast, the deposition of MPO in the subepithelial areas has been observed in some of these cases [28,29], and dual positivity for serum anti-PLA_2_R antibody and MPO-ANCA has also been reported [30]. We have previously reported two patients with MPO-ANCA-associated vasculitis, in whom a renal pathological diagnosis of ANCA-associated glomerulonephritis with MN lesions was established, and both MPO and PLA_2_R were detected in the glomerular deposits [31]. The patient had long-standing serological positivity for MPO-ANCA, whereas serum PLA_2_R antibodies were negative. MPO, which is released from activated neutrophils, is highly cationic and is therefore trapped by the glomerular basement membrane. Although it remains unclear whether immune complexes are formed in situ or in circulation, it has been suggested that persistent stimulation by MPO and MPO-ANCA induces hidden antigens of PLA_2_R present in podocytes to be aberrantly expressed as reactive targets of autoantibodies. There were glomerular PLA_2_R deposits in the absence of circulating anti-PLA_2_R antibodies. In this context, the “kidney as a sink” hypothesis (that is, circulating antibodies against PLA_2_R are not detected at the early phase but become detectable only after the capacity of the kidney is surpassed) was suggested [32]. Indeed, the positivity rate of glomerular PLA_2_R deposits was higher than that of circulating anti-PLA_2_R antibodies [33]. A case of proteinase 3 (PR3)-ANCA-associated vasculitis with MN, in which co-localization of PR3 and IgG along the glomerular capillary walls was observed, was recently reported [34]. The involvement of known MN target antigens in these cases should be investigated in future studies.

Several studies have also reported patients with PLA_2_R and other antigen dual-positive MN. For example, glomerular PLA_2_R has been reported to frequently overlap with the hepatitis B surface antigen in patients with hepatitis B virus-associated MN [35]. In these patients, favorable outcomes were achieved after antiviral therapy, suggestive of secondary MN [36]. Exostosin (EXT) 1/2 is a putative antigen of MN associated with autoimmune diseases, especially systemic lupus erythematosus and mixed connective tissue disease, and EXT-positive MN accounts for 5–10% [8]. Circulating anti-EXT 1/2 antibodies have not been identified, and EXT 1/2-positive MN showed IgG1 predominant glomerular deposition [37]. This type of MN was originally believed to occur only in patients who were PLA_2_R-negative [37]. However, a recent study reported that approximately 5% of patients with PLA_2_R-positive MN showed glomerular positive staining of EXT and that patients with PLA_2_R and EXT dual positivity have features of secondary MN, including C3 hypocomplementemia and IF full-house staining [38]. Thus, aberrant PLA_2_R expression may be induced during disease progression in secondary EXT-associated MN. In contrast, glomerular EXT 1/2 deposition with positive IgG4 staining has been reported in patients with both PLA_2_R-positive and THSD7A-positive MN; glomerular EXT1/2 deposition in these cases is believed to occur as a secondary phenomenon [39,40]. Patients with PLA_2_R and semaphorin 3 B (SEMA3B; identified in 2020 and observed mostly in the pediatric age group [41]) dual positivity and patients with concurrent glomerular PLA_2_R and protocadherin 7 (PCDH7; identified in 2021, accompanied by reduced glomerular complement deposition [42]) deposits have also been reported [43,44].

The incidence of dual antigen-positive MN seems to be comparable to that of some newly identified antigen-associated MN, which is reportedly 1–2% [8]. Therefore, it is reasonable to suggest that more attention should be given to this condition. However, whether the antigens emerge concomitantly or sequentially has not yet been described in detail. In this regard, we introduce some cases from our own experience in which the transition of patient responses to MN antigens was revealed by repeated examination.

## 3. Case 1: NELL1-Associated MN Showing Positive Conversion of Serum Anti-PLA_2_R Antibody [14]

**Figure 1:**
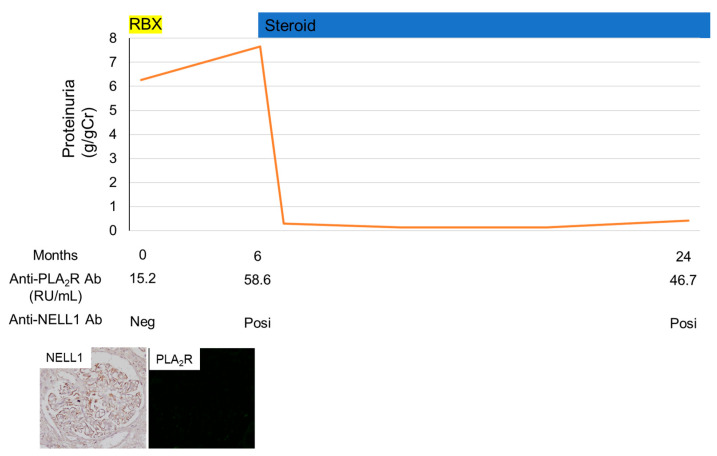
Clinical course of Case 1 [14]. Immunoperoxidase staining of the patient’s renal biopsy (RBX) tissue revealed positivity for neural epidermal growth factor-like 1 protein (NELL1) on the glomerular capillary walls (**left** panel). Immunofluorescence staining for the phospholipase A2 receptor (PLA_2_R, **right** panel) was negative. Original magnification of both panels: 400×. Serum anti-NELL1 antibodies and anti-PLA2R antibodies were assessed by Western blotting as previously described [25] and an enzyme-linked immunosorbent assay kit (EUROIMMUN AG, Lübeck, Germany) in accordance with the manufacturer’s instructions (positive cutoff value, 20 RU/mL; borderline, 14–20 RU/mL), respectively.

A man in his early 70s presented with nephrotic syndrome, and renal biopsy yielded a diagnosis of idiopathic NELL1-associated MN with predominant IgG1 deposition. Circulating anti-NELL1 antibodies were not detected, whereas immunoperoxidase staining revealed glomerular NELL1 deposits. Serum anti-PLA_2_R antibody was borderline, and IF staining for PLA_2_R was negative. Interestingly, the serum obtained approximately half a year after biopsy showed definitive reactivity against NELL1 and PLA_2_R. Corticosteroid treatment was initiated as recommended in the Japanese guidelines [45], which improved the patient’s nephrotic syndrome. Serum obtained after the initiation of steroid therapy showed decreased reactivity against NELL1 and decreased anti-PLA_2_R antibody levels, although both remained positive (Figure 1).

This case report provides valuable information regarding dual antigen-positive MN. First, the serological diagnosis could change depending on the timing of sample collection for analysis; this case might have been diagnosed as PLA_2_R-associated MN if a serological approach alone was performed at the later disease phase. Second, serum PLA_2_R antibody has been reported to directly induce structural changes in and the apoptosis of podocytes [46], and its level has been proposed as a useful marker to predict the disease activity of MN [3], even in patients with dual antigen-positive MN [26]. However, the clinical course of this case suggests that the significance of serum PLA_2_R antibodies in patients with dual antigen-positive MN should be re-evaluated in future studies involving a larger number of participants.

## 4. Case 2: Monoclonal Gammopathy of Renal Significance (MGRS) Presenting as MN Showing Sequential Increase in Glomerular THSD7A Deposits

Atypical MN accompanied by monoclonal Ig deposition, also referred to as monoclonal Ig deposition disease associated with membranous features or proliferative glomerulonephritis with monoclonal Ig deposits and predominant membranous features, is now considered a pathological condition of MGRS [47,48,49]; the target antigens for MN-lesions in these cases remain to be clarified. A patient with IgG3-kappa-type MN experienced recurrence after kidney transplantation [50]. In this patient, glomerular PLA_2_R deposits were co-localized with IgG3, and IgG3-kappa-restricted serum anti-PLA_2_R antibody became undetectable following effective treatment with rituximab, thereby suggesting a pathogenic role for the patient’s anti-PLA_2_R antibody.

We have previously reported a patient with IgM lambda monoclonal gammopathy and MN with monoclonal IgM lambda deposition in whom histological changes were observed on repeat renal biopsy [51]. The first renal biopsy showed solitary IgM lambda deposition without IgG deposition, whereas a repeat biopsy, performed approximately 2 years later because of exacerbation of nephrotic syndrome, showed diminished IgM staining and positive staining for IgG (predominantly IgG2, accompanied by a lesser degree of IgG4) without light chain restriction in routine IF staining using fresh frozen tissue sections. However, immunoperoxidase staining after an antigen retrieval procedure using formalin-fixed, paraffin-embedded tissue sections demonstrated strong IgM deposition in the repeat biopsy tissue, raising the possibility that IgM deposition was challenging to detect due to the deposition of polyclonal IgG, which interferes with the immunoreactivity of the anti-IgM antibody. Two infusions of rituximab (375 mg/m^2^ per administration) were administered, which improved both hematological and renal abnormalities.

**Figure 2:**
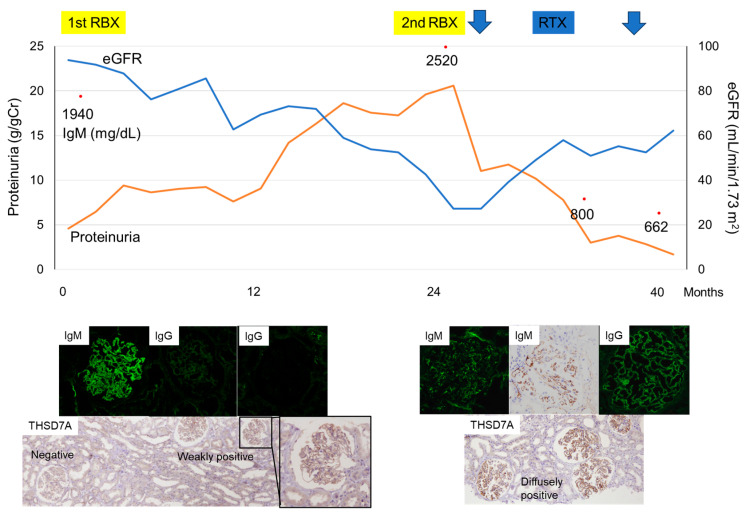
Clinical course of Case 2 [51]. Immunofluorescence (IF) staining of the patient’s first renal biopsy (RBX) tissue showed lambda light-chain-restricted strong immunoglobulin (Ig)M deposition on the glomerular capillary walls. IF IgG staining of freshly frozen tissue sections (left) and formalin-fixed, paraffin-embedded tissue (FFPE) sections was negative. The second RBX tissue sample showed positive IF IgG staining without light chain restriction. Diminished IgM staining was observed by IF staining of freshly frozen tissue sections; however, immunoperoxidase staining of FFPE sections demonstrated strong IgM deposition. Immunostaining for thrombospondin type-1 domain-containing 7A (THSD7A) showed weak, focal, and segmental immunoreactivity in the glomerular capillary walls of the first RBX tissue (A higher magnified image of one glomerulus is highlighted by black rectangle), whereas strong and diffuse positive staining patterns were observed in the second RBX tissue. Original magnification: IgM and IgG staining, 400×; THSD7A staining, 100×. The patient’s serum IgM level was decreased and estimated glomerular filtration (eGFR) level was increased following twice infusions of rituximab (RTX, 375 mg/m^2^ per administration). Blue arrows indicate the timing of RTX administration.

Furthermore, immunostaining for THSD7A showed a diffuse positive staining pattern in the repeat biopsy tissue. However, only focal and segmental immunoreactivity in the glomerular capillary wall was observed in the first biopsy tissue (Figure 2). Thus, the patient would have been diagnosed not with MGRS but with the commonly observed THSD7A-asssociated MN with polyclonal IgG deposition if histological examination was performed only at a late phase of the disease and in a routine manner. Although the first MN antigen in the patient to which the monoclonal IgM lambda antibody reacted remained undetermined, it was suggested that exposure to THSD7A epitopes and autoantibody production against them occurred secondarily during the patient’s clinical course.

We have recently reported an interesting case supporting the above scenario; in this case, histological transition from minimal change disease (MCD) to THSD7A-associated MN with IgG4 predominant glomerular deposition occurred during long-term steroid treatment [52]. Thus, there seems to be another possibility that long-standing podocyte injury during the clinical course of MCD, including the usage of cytotoxic drugs, could induce THSD7A expression, thereby leading to autoantibody production, at least in susceptible individuals. Repeat renal biopsy is rarely performed in patients with MCD or MN, but it is possible that there are more undiagnosed cases that develop a histological transition between these two diseases than we now consider.

## 5. Future Perspectives

Although the data on the characteristic clinical and pathological manifestations of dual antigen-positive MN are limited, our survey of the papers has revealed the following three points: (1) dual antigen-positive MN is at least somewhat present, (2) the diagnosis could change depending on the timing of sample collection for analysis in some of the cases, and (3) these cases might not be accurately diagnosed if only routine examination was performed only at a late phase of the disease. With regard to therapy, differences in response to treatment between dual antigen-positive MN cases and other usual MN cases are of interest, but information on this point is quite limited, and no established therapeutic regimens are available for these patients for now. Although approximately 30% of patients with MN achieve spontaneous remission, some patients who continue to have nephrotic syndrome develop end-stage kidney disease [53]. Clinical trials have shown the effectiveness of two doses of rituximab [54,55], which was also performed in our previously reported case (Case 2 in this manuscript) [51]. Recent international guidelines recommend rituximab treatment as an induction therapy for MN [6]. Further accumulation of these cases is required to obtain detailed clinicopathological information and appropriate therapeutic strategies. In the choice of treatment, however, it should be stressed that the distinction between whether MN is idiopathic or secondary is the most important issue regardless of whether the case is dual antigen-positive, single antigen-positive, or antigen-negative. In secondary MN, the treatment for the primary condition, such as malignancy, infection, or the use of drugs (i.e., discontinuation of the causative drugs), should be given the highest priority. Rituximab use should be considered afterwards.

As summarized in Table 1, the majority of the dual antigen-positive cases were positive for PLA_2_R and another antigen, indicating the high prevalence of PLA_2_R-positive cases and the ease of evaluating glomerular PLA_2_R deposits and serum corresponding antibodies. To our knowledge, there has been no reported case of dual “foreign” antigen-positive MN. In this regard, we propose that the second MN antigen is an endogenous protein of podocytes that is aberrantly exposed during disease processes targeting the first MN antigen; however, this hypothesis should be verified in the future. Furthermore, whether there are cases of three or more antigen-positive MN should be carefully investigated.

IF staining of IgG subclasses is widely performed in cases of dual antigen-positive MN. The results obtained have been regarded as useful for the classification of MN (idiopathic or secondary); however, they have only been evaluated qualitatively. Qualitative evaluation is affected by several factors, including the affinity of the antibodies used for detection; therefore, a wide array of quantitative evaluation methods is desired in the near future. LCM/MS, which plays a crucial role in the discovery of novel target antigens for MN, provides further information about the glomerular deposition of IgG subclasses and complement proteins [56,57]. The technique of the elution of bound antibodies from the tissue is traditional [58] but is still used now [42,59]. Although a comparison of the results obtained from qualitative and quantitative methods has not been well investigated, it might provide novel insights into our understanding of MN pathogenesis.

## 6. Concluding Remarks

Recent technical advances have enabled the identification of target antigens for MN in a significant proportion of patients. A novel framework for the classification of MN has been proposed, and some researchers have proposed a serology-based approach for patients suspected of having MN in the absence of a histological diagnosis [3]. This classification approach is based on the assumption that only one target antigen is detected in each patient. However, some patients show dual antigen-positive MN, at least to some extent, as described in this manuscript. Furthermore, the status of the positivity for MN antigens may change over time, suggesting that strict caution should be paid in making the precise diagnosis. Their clinicopathological characteristics are not well documented, and therapeutic choices mainly rely on thorough case reports. Therefore, further accumulation of cases is required, and greater awareness of this previously underestimated condition is crucial.

## Figures and Tables

**Table 1:** Characteristics of the target antigens involved in cases of dual antigen-positive MN.

Target Antigen	Year Reported	Podocyte Expressed?	Circulating Autoantibody	Associated Conditions *	Incidence *	Renal Pathological Features *	Another Antigen Involved in Dual Antigen-Positive Cases
PLA_2_R	2009	Yes	Yes	None	50–80%	IgG4 predominant	THSD7A, NELL1, EXT 1/2, SEMA3B, PCDH7, MPO, HBsAg
THSD7A	2014	Yes	Yes	(Occasionally) malignancy	1–5%	IgG4 predominant	PLA_2_R, EXT 1/2
NELL1	2019	No	Yes	Malignancy, autoimmune, drugs	Approximately 10%	IgG1 predominant, subepithelial immune deposits may be segmental	PLA_2_R
EXT 1/2	2019	Yes	No	Autoimmune	5–10%	IgG1 predominant, proliferative features, IgA/IgM deposition, and mesangial deposits may be present	PLA_2_R, THSD7A

* Restricted to single antigen-positive cases. EXT, exostosin; HBsAg, hepatitis B surface antigen; Ig, immunoglobulin; MN, membranous nephropathy; MPO, myeloperoxidase; NELL1, neural epidermal growth factor-like 1 protein; PCDH7, protocadherin 7; PLA_2_R, phospholipase A2 receptor; SEMA3B, semaphorin 3 B; THSD7A, thrombospondin type-1 domain-containing 7A.

## Data Availability

The data presented in this manuscript are available upon request from the corresponding author.
